# 3a*H*,4*H*,5*H*,8*H*,9*H*,9a*H*-Cyclo­octa­[*d*][1,3]dioxole-2-thione

**DOI:** 10.1107/S2414314624010198

**Published:** 2024-11-08

**Authors:** Dieter Schollmeyer, Claudia Kammler, Heiner Detert

**Affiliations:** a University of Mainz, Department of Chemistry, Duesbergweg 10-14, 55099 Mainz, Germany; Goethe-Universität Frankfurt, Germany

**Keywords:** crystal structure, sulfur, heterocycles

## Abstract

The thio­nocarbonate of *trans*-cyclo­octenediol, C_9_H_12_O_2_S, crystallizes with a 9/1 disorder in the position of the *R,R* and *S,S*-enanti­omers. As a result of *trans*-annulation, both rings adopt a twist conformation.

## Graphical abstract

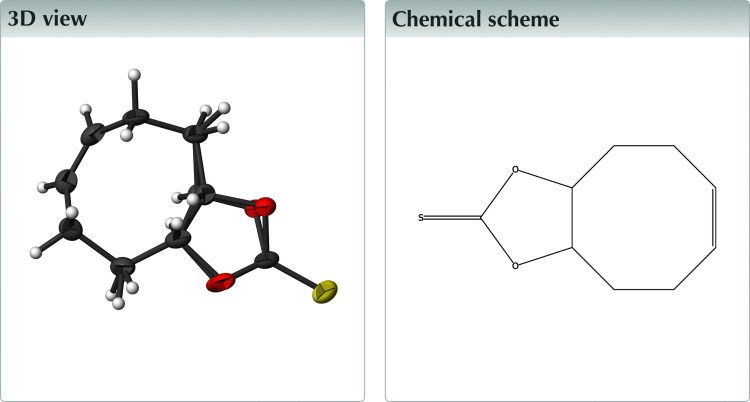

## Structure description

**Figure 1:**
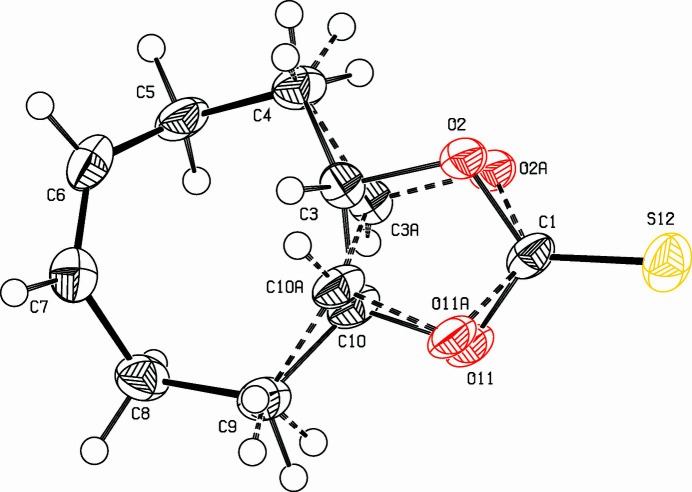
View of the title compound. Displacement ellipsoids are drawn at the 50% probability level. Bonds involving the lower occupied sites are drawn with broken tubes.

**Figure 2:**
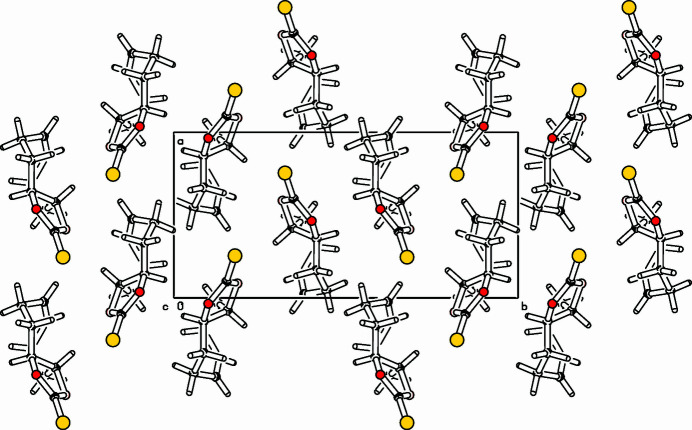
Part of the packing diagram. View along the *c*-axis. Minor occupied sites are omitted for clarity.

Cyclic thio­nocarbonates, 1,3-dioxolan-2-thio­nes, are important inter­mediates for several transformations (Klein *et al.*, 2022 ▸; Rizzo & Trauner, 2018 ▸). Outstanding in this context is the Corey–Winter reaction, a reductive desulfuration with fragmentation of the heterocycle leading to alkenes (Corey *et al.* 1965 ▸). This method allows *cis*–*trans* isomerizations of alkenes and the synthesis of strained compounds (Paquette *et al.*, 1975 ▸; Daub *et al.*, 1972 ▸). As part of our inter­est in strained hydro­carbons (Detert & Meier, 1997*a* ▸,*b* ▸), the title compound was prepared as a precursor for the ‘labile’ 1,5-cyclo­octa­diene (Ziegler & Wilms, 1950 ▸). The racemate crystallizes with disorder, the positions of the title mol­ecule are filled in a 9/1 ratio with *S,S*- and *R,R*-enanti­omers (Fig. 1 ▸). As a result of *trans*-annulation located on C3,C10, both rings adopt a twist conformation. Furthermore, the eight-membered ring forms two planes, the olefinic unit (C5,C6,C7,C8) with maximum deviation of 0.008 (7) Å from the mean plane and the aliphatic part (C4,C5,C8,C9), maximum deviation 0.051 (8) Å. Then angle between the mean planes amounts to 67.5 (5)°. The cyclic thio­nocarbonate is nearly planar, C3 lies slightly above the mean plane [0.092 (8) Å] and C10 similarly below [0.094 (2) Å]. The exocyclic sulfur atom deviates from this plane by just 0.01 (2) Å. The packing is shown in Fig. 2 ▸.

## Synthesis and crystallization

(5*Z*-1,2-*trans*)-Cyclo­oct-5-ene-1,2-diol (3.00 g, 0.02 mol, 1.00 eq), 4-dimethylaminopyridine (DMAP; 5.86 g, 0.05 mol, 2.40 eq), pyridine (32.24 ml, 0.40 mol) and di­chloro­methane (40.00 ml) were placed in a flask with a magnetic stirrer under a nitro­gen atmosphere. The mixture was cooled in ice–water, while a solution of thio­phosgene (2.76 g, 0.02 mol, 1.20 eq) in di­chloro­methane (20.00 ml) was added over 1 h, after one additional hour the reaction mixture was allowed to warm up to room temperature and was stirred for 16 h. The solvent was removed by distillation. The reaction mixture was diluted with a saturated sodium chloride solution (40.00 ml). The aqueous phase was separated and extracted with ethyl acetate (4 × 50.00 ml). The combined organic phases were dried over magnesium sulfate. The mixture was filtered to remove the magnesium sulfate and the solvent was removed under reduced pressure. The crude product was purified by column chromatography (EtOAc:cyclo­hexane = 1:3). The light-orange solid obtained was recrystallized from cyclo­hexane solution. (*Z*)-3a,4,5,8,9,9a-Hexa­hydro­cyclo­octa­[*d*][1,3]dioxole-2-thione (0.38 g, 2.00 mmol, 10%) was obtained as colorless crystals. TLC: *R*_f_ = 0.46 (EtOAc:Cyclo­hexane = 1:3). Melting range: (EtOAc) = 127–132°C. ESI–HRMS (pos.): calc. [C_9_H_12_O_2_S]^+^: *m*/*z* = 185.0631, found: *m*/*z* = 185.0632. ^1^H-NMR: (300 MHz, CDCl_3_); δ [p.p.m.] = 5.70–5.58 (*m*, 2H, 5-CH, 6-CH), 4.72–4.62 (*m*, 2H, 2-CH, 9-CH), 2.36–2.23 (*m*, 4H, 4-CH_2_, 7-CH_2_), 2.23–2.13 (*m*, 2H, 3-CH_2_, 8-CH_2_), 1.77–1.61 (*m*, 2H, 3-CH_2_, 8-CH_2_). ^13^C-NMR: (75 MHz, CDCl_3_); δ [p.p.m.] = 191.51 (1 C, C=S), 129.21 (2 C, 5-CH, 6-CH), 87.27 (2 C, 2-CH, 9-CH), 29.23 (2 C, 3-CH_2_, 8-CH_2_), 20.66 (2 C, 4-CH_2_, 7-CH_2_). The assignment of H- and C-signals is based on HH-Cosy, HMBC– and HSQC– experiments. IR: 3018 (*w*), 2952 (*w*), 1449 (*w*), 1322 (*s*), 1257 (*s*), 1039 (*s*), 965 (*s*), 878 (*w*), 783 (*m*), 595 (*w*) cm^−1^.

## Refinement

Crystal data, data collection and structure refinement details are summarized in Table 1 ▸. Hydrogen atoms were placed at calculated positions and were refined in the riding-model approximation with C_aromatic_–H = 0.95 Å, C_methyl­ene_–H = 0.99 Å, and with *U*_iso_(H) = 1.2 *U*_eq_(C). The site occupation factors were kept fixed at 0.9 and 0.1 for the disordered sites. The displacement parameters of the disordered C and O atoms were constrained to be equal for the corresponding sites. The absolute structure could not be determined reliably.

## Supplementary Material

Crystal structure: contains datablock(s) I, global. DOI: 10.1107/S2414314624010198/bt4155sup1.cif

Structure factors: contains datablock(s) I. DOI: 10.1107/S2414314624010198/bt4155Isup2.hkl

Supporting information file. DOI: 10.1107/S2414314624010198/bt4155Isup3.cml

CCDC reference: 2391879

Additional supporting information:  crystallographic information; 3D view; checkCIF report

## Figures and Tables

**Table 1:** Experimental details

Crystal data	
Chemical formula	C_9_H_12_O_2_S
*M*_r_	184.25
Crystal system, space group	Orthorhombic, *Pna*2_1_
Temperature (K)	120
*a*, *b*, *c* (Å)	7.5555 (9), 15.7119 (17), 7.4892 (8)
*V* (Å^3^)	889.05 (17)
*Z*	4
Radiation type	Mo *K*α
μ (mm^−1^)	0.32
Crystal size (mm)	0.57 × 0.09 × 0.03

Data collection	
Diffractometer	Stoe *IPDS* 2T
Absorption correction	Integration
*T*_min_, *T*_max_	0.898, 0.989
No. of measured, independent and observed [*I* > 2σ(*I*)] reflections	3713, 1963, 1439
*R*_int_	0.055
(sin θ/λ)_max_ (Å^−1^)	0.660

Refinement	
*R*[*F*^2^ > 2σ(*F*^2^)], *wR*(*F*^2^), *S*	0.070, 0.159, 1.10
No. of reflections	1963
No. of parameters	121
No. of restraints	6
H-atom treatment	H-atom parameters constrained
Δρ_max_, Δρ_min_ (e Å^−3^)	0.32, −0.29
Absolute structure	Classical Flack method preferred over Parsons because s.u. lower.
Absolute structure parameter	0.3 (3)

Computer programs: *X-AREA WinXpose*, *Recipe* and *Integrate* (Stoe & Cie, 2020 ▸), *SHELXT2014* (Sheldrick, 2015*a* ▸), *SHELXL2019/2* (Sheldrick, 2015*b* ▸) and *PLATON* (Spek, 2020 ▸).

## full crystallographic data

### 3aH,4H,5H,8H,9H,9aH-Cycloocta[d][1,3]dioxole-2-thione Crystal data

**Table:**

C_9_H_12_O_2_S	*D*_x_ = 1.377 Mg m^−^^3^
*M**_r_* = 184.25	Mo *K*α radiation, λ = 0.71073 Å
Orthorhombic, *P**n**a*2_1_	Cell parameters from 3621 reflections
*a* = 7.5555 (9) Å	θ = 3.0–28.2°
*b* = 15.7119 (17) Å	µ = 0.32 mm^−^^1^
*c* = 7.4892 (8) Å	*T* = 120 K
*V* = 889.05 (17) Å^3^	Needle, colorless
*Z* = 4	0.57 × 0.09 × 0.03 mm
*F*(000) = 392	

### 3aH,4H,5H,8H,9H,9aH-Cycloocta[d][1,3]dioxole-2-thione Data collection

**Table:**

Stoe IPDS 2T diffractometer	1963 independent reflections
Radiation source: sealed X-ray tube, 12x0.4mm long-fine focus	1439 reflections with *I* > 2σ(*I*)
Detector resolution: 6.67 pixels mm^-1^	*R*_int_ = 0.055
rotation method, ω scans	θ_max_ = 28.0°, θ_min_ = 3.0°
Absorption correction: integration	*h* = −9→9
*T*_min_ = 0.898, *T*_max_ = 0.989	*k* = −20→20
3713 measured reflections	*l* = −9→8

### 3aH,4H,5H,8H,9H,9aH-Cycloocta[d][1,3]dioxole-2-thione Refinement

**Table:**

Refinement on *F*^2^	Hydrogen site location: inferred from neighbouring sites
Least-squares matrix: full	H-atom parameters constrained
*R*[*F*^2^ > 2σ(*F*^2^)] = 0.070	*w* = 1/[σ^2^(*F*_o_^2^) + (0.0349*P*)^2^ + 2.2293*P*] where *P* = (*F*_o_^2^ + 2*F*_c_^2^)/3
*wR*(*F*^2^) = 0.159	(Δ/σ)_max_ = 0.002
*S* = 1.10	Δρ_max_ = 0.32 e Å^−^^3^
1963 reflections	Δρ_min_ = −0.29 e Å^−^^3^
121 parameters	Absolute structure: Classical Flack method preferred over Parsons because s.u. lower.
6 restraints	Absolute structure parameter: 0.3 (3)
Primary atom site location: dual	

### 3aH,4H,5H,8H,9H,9aH-Cycloocta[d][1,3]dioxole-2-thione Special details

**Table:**

Geometry. All esds (except the esd in the dihedral angle between two l.s. planes) are estimated using the full covariance matrix. The cell esds are taken into account individually in the estimation of esds in distances, angles and torsion angles; correlations between esds in cell parameters are only used when they are defined by crystal symmetry. An approximate (isotropic) treatment of cell esds is used for estimating esds involving l.s. planes.

### 3aH,4H,5H,8H,9H,9aH-Cycloocta[d][1,3]dioxole-2-thione Fractional atomic coordinates and isotropic or equivalent isotropic displacement parameters (Å^2^)

**Table:**

	*x*	*y*	*z*	*U*_iso_*/*U*_eq_	Occ. (<1)
C1	0.5985 (9)	0.3473 (4)	0.6938 (9)	0.0291 (14)	
O2	0.5867 (7)	0.3159 (3)	0.5280 (6)	0.0292 (12)	0.9
C3	0.4227 (10)	0.3437 (5)	0.4437 (10)	0.0267 (15)	0.9
H3	0.335024	0.296075	0.443307	0.032*	0.9
C4	0.4633 (10)	0.3704 (5)	0.2539 (10)	0.0324 (16)	
H4A	0.495129	0.319495	0.183013	0.039*	0.9
H4B	0.566653	0.409199	0.254135	0.039*	0.9
H4C	0.448687	0.307895	0.247867	0.039*	0.1
H4D	0.575352	0.386047	0.193598	0.039*	0.1
C5	0.3070 (9)	0.4151 (5)	0.1658 (9)	0.0350 (16)	
H5A	0.293459	0.472383	0.219282	0.042*	
H5B	0.332900	0.422742	0.037191	0.042*	
C6	0.1361 (10)	0.3678 (4)	0.1850 (10)	0.0357 (17)	
H6	0.107377	0.326913	0.096048	0.043*	
C7	0.0227 (9)	0.3789 (4)	0.3170 (11)	0.0334 (16)	
H7	−0.080012	0.344029	0.318413	0.040*	
C8	0.0442 (10)	0.4430 (4)	0.4663 (10)	0.0349 (16)	
H8A	−0.073915	0.455640	0.517033	0.042*	
H8B	0.091387	0.496579	0.415429	0.042*	
C9	0.1672 (9)	0.4140 (5)	0.6185 (9)	0.0335 (17)	
H9A	0.150571	0.452070	0.722425	0.040*	0.9
H9B	0.133462	0.355718	0.655515	0.040*	0.9
H9C	0.094452	0.382082	0.705681	0.040*	0.1
H9D	0.211017	0.465622	0.679986	0.040*	0.1
C10	0.3590 (11)	0.4144 (5)	0.5659 (10)	0.0307 (17)	0.9
H10	0.390122	0.470873	0.512854	0.037*	0.9
O11	0.4647 (7)	0.4000 (4)	0.7264 (6)	0.0317 (13)	0.9
S12	0.7542 (3)	0.32268 (11)	0.8348 (3)	0.0398 (4)	
O2A	0.635 (6)	0.375 (3)	0.523 (6)	0.0292 (12)	0.1
C3A	0.465 (6)	0.400 (4)	0.446 (4)	0.0267 (15)	0.1
H3A	0.449620	0.463421	0.452080	0.032*	0.1
C10A	0.335 (7)	0.357 (4)	0.570 (6)	0.0307 (17)	0.1
H10A	0.297608	0.300001	0.521916	0.037*	0.1
O11A	0.430 (3)	0.347 (3)	0.740 (5)	0.0317 (13)	0.1

### 3aH,4H,5H,8H,9H,9aH-Cycloocta[d][1,3]dioxole-2-thione Atomic displacement parameters (Å^2^)

**Table:**

	*U*^11^	*U*^22^	*U*^33^	*U*^12^	*U*^13^	*U*^23^
C1	0.037 (4)	0.032 (3)	0.018 (3)	−0.007 (3)	0.000 (3)	0.001 (3)
O2	0.031 (3)	0.035 (3)	0.021 (3)	0.005 (2)	0.000 (2)	−0.001 (2)
C3	0.027 (4)	0.027 (3)	0.027 (4)	0.000 (3)	0.000 (3)	0.004 (3)
C4	0.040 (4)	0.035 (4)	0.022 (4)	0.003 (3)	0.005 (3)	0.003 (3)
C5	0.046 (4)	0.043 (4)	0.016 (3)	0.005 (4)	0.001 (3)	0.000 (3)
C6	0.046 (4)	0.033 (3)	0.028 (4)	0.002 (3)	−0.013 (4)	−0.002 (3)
C7	0.034 (3)	0.029 (3)	0.038 (5)	0.000 (3)	−0.005 (4)	0.001 (3)
C8	0.036 (4)	0.035 (3)	0.034 (4)	0.010 (3)	0.004 (3)	0.001 (3)
C9	0.040 (4)	0.036 (4)	0.025 (4)	0.006 (3)	0.003 (3)	−0.001 (3)
C10	0.045 (5)	0.029 (4)	0.019 (4)	0.005 (4)	−0.001 (3)	0.000 (3)
O11	0.041 (3)	0.037 (3)	0.017 (3)	0.004 (3)	−0.003 (2)	−0.005 (2)
S12	0.0412 (8)	0.0489 (9)	0.0293 (8)	−0.0041 (10)	−0.0065 (9)	0.0080 (10)
O2A	0.031 (3)	0.035 (3)	0.021 (3)	0.005 (2)	0.000 (2)	−0.001 (2)
C3A	0.027 (4)	0.027 (3)	0.027 (4)	0.000 (3)	0.000 (3)	0.004 (3)
C10A	0.045 (5)	0.029 (4)	0.019 (4)	0.005 (4)	−0.001 (3)	0.000 (3)
O11A	0.041 (3)	0.037 (3)	0.017 (3)	0.004 (3)	−0.003 (2)	−0.005 (2)

### 3aH,4H,5H,8H,9H,9aH-Cycloocta[d][1,3]dioxole-2-thione Geometric parameters (Å, º)

**Table:**

C1—O11A	1.32 (3)	C6—H6	0.9500
C1—O11	1.330 (8)	C7—C8	1.515 (10)
C1—O2	1.339 (8)	C7—H7	0.9500
C1—O2A	1.38 (5)	C8—C9	1.539 (10)
C1—S12	1.627 (7)	C8—H8A	0.9900
O2—C3	1.458 (8)	C8—H8B	0.9900
C3—C4	1.514 (10)	C9—C10	1.502 (10)
C3—C10	1.517 (10)	C9—C10A	1.60 (7)
C3—H3	1.0000	C9—H9A	0.9900
C4—C3A	1.51 (3)	C9—H9B	0.9900
C4—C5	1.524 (10)	C9—H9C	0.9900
C4—H4A	0.9900	C9—H9D	0.9900
C4—H4B	0.9900	C10—O11	1.461 (8)
C4—H4C	0.9900	C10—H10	1.0000
C4—H4D	0.9900	O2A—C3A	1.46 (3)
C5—C6	1.497 (10)	C3A—C10A	1.52 (3)
C5—H5A	0.9900	C3A—H3A	1.0000
C5—H5B	0.9900	C10A—O11A	1.47 (3)
C6—C7	1.320 (10)	C10A—H10A	1.0000
			
O11—C1—O2	110.5 (6)	C7—C8—H8A	108.6
O11A—C1—O2A	116 (3)	C9—C8—H8A	108.6
O11A—C1—S12	122 (2)	C7—C8—H8B	108.6
O11—C1—S12	125.4 (5)	C9—C8—H8B	108.6
O2—C1—S12	124.2 (5)	H8A—C8—H8B	107.6
O2A—C1—S12	122.2 (17)	C10—C9—C8	112.8 (6)
C1—O2—C3	110.3 (5)	C8—C9—C10A	118.6 (17)
O2—C3—C4	108.5 (6)	C10—C9—H9A	109.0
O2—C3—C10	103.2 (6)	C8—C9—H9A	109.0
C4—C3—C10	115.3 (6)	C10—C9—H9B	109.0
O2—C3—H3	109.8	C8—C9—H9B	109.0
C4—C3—H3	109.8	H9A—C9—H9B	107.8
C10—C3—H3	109.8	C8—C9—H9C	107.7
C3—C4—C5	112.2 (6)	C10A—C9—H9C	107.7
C3A—C4—C5	105.9 (18)	C8—C9—H9D	107.7
C3—C4—H4A	109.2	C10A—C9—H9D	107.7
C5—C4—H4A	109.2	H9C—C9—H9D	107.1
C3—C4—H4B	109.2	O11—C10—C9	108.1 (5)
C5—C4—H4B	109.2	O11—C10—C3	102.1 (6)
H4A—C4—H4B	107.9	C9—C10—C3	117.5 (7)
C3A—C4—H4C	110.5	O11—C10—H10	109.6
C5—C4—H4C	110.5	C9—C10—H10	109.6
C3A—C4—H4D	110.5	C3—C10—H10	109.6
C5—C4—H4D	110.5	C1—O11—C10	111.1 (5)
H4C—C4—H4D	108.7	C1—O2A—C3A	106 (3)
C6—C5—C4	113.4 (6)	O2A—C3A—C4	107 (3)
C6—C5—H5A	108.9	O2A—C3A—C10A	102 (4)
C4—C5—H5A	108.9	C4—C3A—C10A	116 (4)
C6—C5—H5B	108.9	O2A—C3A—H3A	110.4
C4—C5—H5B	108.9	C4—C3A—H3A	110.4
H5A—C5—H5B	107.7	C10A—C3A—H3A	110.4
C7—C6—C5	124.5 (7)	O11A—C10A—C3A	105 (4)
C7—C6—H6	117.8	O11A—C10A—C9	104 (4)
C5—C6—H6	117.8	C3A—C10A—C9	113 (4)
C6—C7—C8	124.9 (6)	O11A—C10A—H10A	111.2
C6—C7—H7	117.6	C3A—C10A—H10A	111.2
C8—C7—H7	117.6	C9—C10A—H10A	111.2
C7—C8—C9	114.5 (6)	C1—O11A—C10A	104 (3)
			
O11—C1—O2—C3	−5.2 (7)	S12—C1—O11—C10	174.4 (5)
S12—C1—O2—C3	174.1 (5)	C9—C10—O11—C1	138.8 (6)
C1—O2—C3—C4	136.5 (6)	C3—C10—O11—C1	14.3 (8)
C1—O2—C3—C10	13.7 (7)	O11A—C1—O2A—C3A	2 (5)
O2—C3—C4—C5	−169.9 (6)	S12—C1—O2A—C3A	−173 (3)
C10—C3—C4—C5	−54.7 (9)	C1—O2A—C3A—C4	−140 (3)
C3—C4—C5—C6	−49.4 (8)	C1—O2A—C3A—C10A	−17 (5)
C3A—C4—C5—C6	−87 (2)	C5—C4—C3A—O2A	−167 (3)
C4—C5—C6—C7	91.1 (8)	C5—C4—C3A—C10A	80 (4)
C5—C6—C7—C8	2.1 (11)	O2A—C3A—C10A—O11A	26 (6)
C6—C7—C8—C9	−81.3 (9)	C4—C3A—C10A—O11A	142 (4)
C7—C8—C9—C10	72.8 (8)	O2A—C3A—C10A—C9	139 (4)
C7—C8—C9—C10A	35 (3)	C4—C3A—C10A—C9	−105 (4)
C8—C9—C10—O11	170.6 (6)	C8—C9—C10A—O11A	175 (3)
C8—C9—C10—C3	−74.7 (9)	C8—C9—C10A—C3A	61 (4)
O2—C3—C10—O11	−15.9 (7)	O2A—C1—O11A—C10A	15 (5)
C4—C3—C10—O11	−134.1 (6)	S12—C1—O11A—C10A	−170 (3)
O2—C3—C10—C9	−134.0 (6)	C3A—C10A—O11A—C1	−25 (6)
C4—C3—C10—C9	107.9 (7)	C9—C10A—O11A—C1	−144 (3)
O2—C1—O11—C10	−6.3 (8)		
